# The Role of Adipokines in Spinal Disease: A Narrative Review

**DOI:** 10.1002/jsp2.70083

**Published:** 2025-06-03

**Authors:** Jake M. McDonnell, Stacey Darwish, Joseph S. Butler, Conor T. Buckley

**Affiliations:** ^1^ National Spinal Injuries Unit Mater Misericordiae University Hospital Dublin Ireland; ^2^ Trinity Centre for Biomedical Engineering Trinity Biomedical Sciences Institute, Trinity College Dublin, the University of Dublin Dublin Ireland; ^3^ Department of Orthopaedics St. Vincent's University Hospital Dublin Ireland; ^4^ School of Medicine University of College Dublin Belfield, Dublin Ireland; ^5^ Discipline of Mechanical, Manufacturing and Biomedical Engineering School of Engineering, Trinity College Dublin, the University of Dublin Dublin Ireland; ^6^ Advanced Materials and Bioengineering Research (AMBER) Centre, Royal College of Surgeons in Ireland & Trinity College Dublin The University of Dublin Dublin Ireland; ^7^ Tissue Engineering Research Group, Department of Anatomy and Regenerative Medicine Royal College of Surgeons in Ireland Dublin Ireland

**Keywords:** adipokines, degeneration, intervertebral disc, leptin, spinal pathology

## Abstract

**Background:**

Adipokines are bioactive molecules secreted by adipose tissue that influence both local and systemic physiological processes. Although adipokine dysregulation has been widely implicated in the pathogenesis of cardiovascular, gastrointestinal, nervous, immune, and musculoskeletal disorders‐ especially osteoarthritis, rheumatoid arthritis, and osteoporosis‐ their involvement in spinal pathology remains comparatively underinvestigated. Recent evidence indicates that certain adipokines may influence spinal disease progression by affecting bone, intervertebral discs, musculature, and neural structures.

**Methods:**

A comprehensive literature review was conducted to evaluate preclinical and clinical studies investigating the role of adipokines in spinal pathology. PubMed and related databases were searched for studies reporting associations between adipokine expression and structural or functional changes in spinal tissues, including vertebral bone, intervertebral discs, paraspinal muscles, spinal ligaments, and the spinal cord. Particular attention was given to mechanistic insights and translational relevance.

**Results:**

The review identified emerging evidence implicating several adipokines, including leptin, adiponectin, resistin, and visfatin, in degenerative and inflammatory changes across various spinal structures. Adipokines were found to influence matrix degradation, bone turnover, muscle atrophy, and neural inflammation through cytokine signaling, oxidative stress, and metabolic dysregulation. However, findings are often heterogeneous and context‐dependent, with limited longitudinal or interventional data.

**Conclusions:**

Adipokines represent a promising yet underexplored avenue in spinal disease research. Their diverse functions in both structural and metabolic regulation highlight their potential as both biomarkers and therapeutic targets. Further mechanistic and clinical studies are needed to elucidate causal relationships and therapeutic efficacy, particularly in degenerative spine disorders.

## Graphical abstract

Adipokines, bioactive molecules secreted by adipose tissue, play a key role in both physiological regulation and disease pathogenesis. While their involvement in various organ systems is well documented, their role in spinal pathology remains underexplored. This review synthesizes current evidence on adipokine influence in spinal disease, examining their effects on bone, intervertebral disc tissue, paraspinal musculature, ligaments, and the spinal cord, while also discussing potential therapeutic interventions.

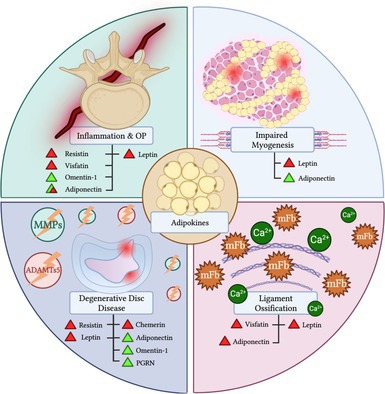

## 1 Introduction

**FIGURE 1:**
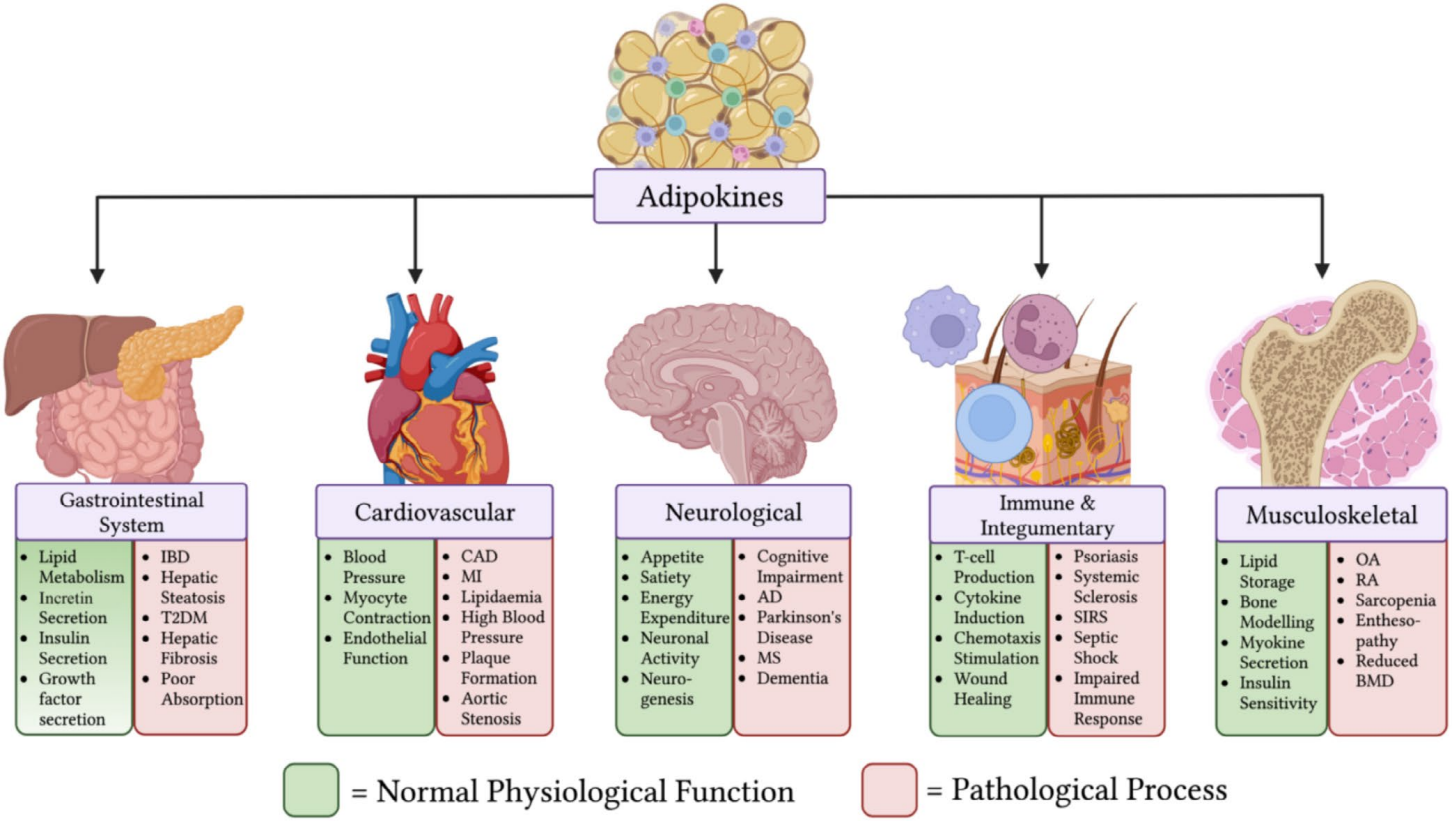
Summary of the role of adipokines in various physiological and pathological processes. (IBD) = inflammatory bowel disease. (T2DM) = type 2 diabetes mellitus. (CAD) = coronary artery disease. (MI) = myocardial infarction. (AD) = Alzheimer's disease. (MS) = multiple sclerosis. (OA) = osteoarthritis. (RA) = rheumatoid arthritis. (BMD) = bone mineral density.

Adipose tissue is integral in the dynamic regulation of various physiological functions. This can be achieved via the release of adipokines, bioactive molecules which act as endocrine hormones exerting paracrine and systemic effects [1]. Leptin was the first adipokine reported in academic literature in 1994 [2]. To date, more than 600 adipokines have been discovered [3]. However, it is important to note that adipose tissue is not a uniform organ and has different secretory profiles depending on location. As such, there is an ever‐evolving list of novel adipokines being discovered, many of whose exact functions currently remain unknown [4]. Some of the most commonly secreted adipokines are adiponectin, leptin, resistin, visfatin, and chemerin, among others [3, 4]. These molecules can act on various organ systems and are involved in diverse physiological processes such as inflammation, hemostasis, and regulation of blood pressure [4, 5]. Despite their pivotal role in normal physiological function, adipokine dysregulation has been highlighted in various disease states, as outlined in Figure 1 [5, 6, 7, 8, 9, 10].

Over‐ and under‐expression of particular adipokines has been shown to be involved in several pathophysiological mechanisms in developing disease states. The majority of published literature pertains to cardiometabolic dysfunction, atherosclerosis, and insulin resistance [7, 8, 9, 10]. Recently, their role in musculoskeletal disease has been reported, including spine‐related disease [6]. However, this evidence remains in its relative infancy. This review outlines the positive and negative influence that adipokines exert on pathological processes in certain aspects of spinal anatomy and disease, including bone, intervertebral discs (IVDs), paraspinal musculature, ligaments, and the spinal cord.

## 2 Influence of Adipokines in Bone‐Related Spinal Pathology

**FIGURE 2:**
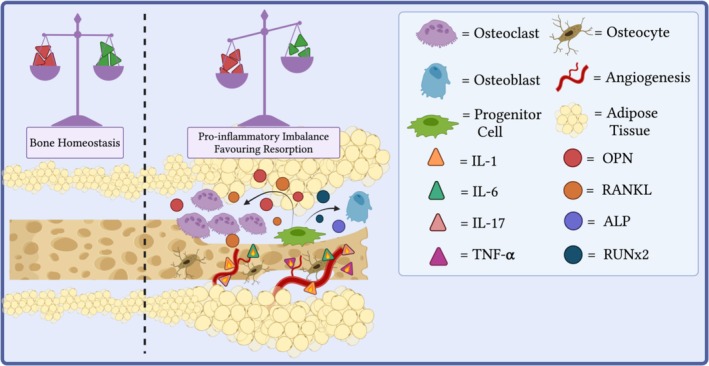
Adipokine dysregulation resulting in a pro‐inflammatory imbalance, leading to bone resorption. (IL) = interleukin. (TNF‐α) = tumor necrosis factor‐alpha. (OPN) = osteopontin. (RANKL) = receptor activator of nuclear factor kappa‐Β ligand. (ALP) = alkaline phosphatase. (RUNx2) = runt‐related transcription factor 2.

The influence of adipokines in bone‐related spinal pathology has been reported in the literature, particularly in relation to degenerative conditions and reduced bone mineral density (BMD) states (Figure 2), ankylosing spondylitis (AS) and spinal deformities [11, 12, 13, 14, 15, 16, 17, 18, 19, 20]. A clinical study by Thommesen et al. [21] highlighted a significant negative correlation between serum resistin levels and BMD in the lumbar spine, corroborated by further studies, and is believed to occur due to resistin's apparent ability to stimulate osteoclastogenesis by increasing levels of inflammatory cytokines and matrix degrading enzymes via the nuclear factor kappa beta (NF‐Kβ) and WNT pathway [22, 23, 24, 25, 26, 27]. However, some studies have contested the link between resistin and decreased BMD, reporting no significant association between serum resistin levels and BMD in osteoarthritis (OA) patients [28].

Comparably, Ma et al. [28] reported that patients with thoracolumbar osteoporotic vertebral compression fractures (OVCF) exhibited the highest serum levels of leptin, IL‐2, IL‐6, and TNF‐α, followed by osteoporotic patients without fractures, compared to healthy controls. Interestingly, the authors also report abnormal levels of proteins responsible for regulating the WNT pathway, which Philip et al. [26] previously noted to be the pathway through which resistin mediated its osteoclastogenic attributes. Thus, similarities exist across studies relating to the influence that adipokines exert on overall BMD. Furthermore, the pro‐catabolic activity of other adipokines in OA cohorts has been reported, including visfatin and chemerin, among others [29, 30, 31, 32]. However, certain associations have been challenged [27, 32]. For example, Yamauchi et al. [32] report, in a cohort of postmenopausal women with vertebral fractures, a positive correlation between plasma leptin concentrations and lumbar BMD on dual‐energy X‐ray absorptiometry (DXA). In contrast, omentin‐1 is an adipokine shown to consistently reduce pathological bone resorption by downregulating the production of pro‐inflammatory factors in TNF‐α activated macrophages, thereby suppressing anti‐osteoblastic and pro‐osteoclastic abilities [31]. Therefore, differences in cohort characteristics may account for discrepancies reported among studies as Araneta et al. [33] highlight significant results are dependent on sex. The authors report that adiponectin was mildly, yet significantly inversely associated with BMD in the lumbar spine and only in female patients. However, adiponectin was associated with vertebral fractures among men only. Therefore, further studies are needed to elucidate the relationship between adipokines, lumbar BMD, degenerative conditions, and the impact of respective sex.

Regarding AS, studies have highlighted a relationship between serum adipokine levels, clinical presentation, and radiographic spinal progression, typically defined by the modified Stoke ankylosing spondylitis score (MSASS). In a study of 111 patients (86 AS vs. 25 healthy controls) by Syrbe et al. [34], mean serum levels of resistin and visfatin were significantly higher. Concerning radiographic progression, Syrbe et al. reported that on multivariate analysis, visfatin > 8 ng/mL was significantly associated with syndesmophyte formation and worsening of MSASS by 2 or greater units. Comparable results were noted by Rademacher et al. [35], who reported that baseline visfatin levels are also associated with MSASS progression at 2 years. Of note, other studies have reported positive correlations between baseline serum adiponectin, leptin, resistin, and either MSASS progression at 2 years or syndesmophyte formation/progression [12, 36]. Similar to studies concerning degenerative conditions, future studies should evaluate discrepancies between genders, as AS is four times more common in males.

Beyond degenerative and inflammatory conditions, the role of adipokines in pediatric spinal deformity has been reported. Wang et al. [37] noted significantly higher serum levels of leptin in adolescent idiopathic scoliosis (AIS patients) compared to controls in a meta‐analysis of 7 studies. Normand et al. [38] depict that in an AIS cohort study of 42 patients, participants with a Cobb angle superior to 25° had higher mean resistin levels compared to controls, while a large cohort study by Zhang et al. [13] highlights that AIS patients had higher serum adiponectin levels compared to controls. However, this only reached statistical significance when comparing AIS osteopenia to controls. Nevertheless, the authors report particularly interesting results. When the concave and convex sides of scoliotic curves were compared, relative adiponectin receptor‐1 (AdipoR1) protein expression levels were shown to be significantly higher in the convex side than the concave side, in addition to RANK, RANKL, and RANKL/OPG levels [13]. This highlights discrepancies between respective sides of spinal deformities, alluding to adipokines possessing the ability to exert local effects. This relationship requires further evaluation as many patients with spinal deformities undergo surgery at some point in their life and despite a lack of robust evidence, preliminary results highlight temporal expression of adipokines after spinal fusion, potentially influencing fusion rates [32]. Nevertheless, further research is needed to clarify the role of adipokines in vertebral disease and how recovery may be affected.

## 3 The Role of Adipokines in Degenerative Disc Disease

Degenerative processes in nucleus pulposus (NP), annulus fibrosus (AF) and cartilaginous endplate (CEP) can result in degenerative disc disease (DDD). DDD represents a common cause of patients presenting with low back pain and neurological deficits and can often require surgical intervention [40]. Akin to bone‐related research, several pre‐clinical and clinical studies demonstrate a strong association between adipokines and the development of DDD [41, 42, 43, 44, 45, 46, 47, 48, 49, 50].

Leptin has been shown to negatively influence matrix synthesis rates, with down‐regulation evident in NP and AF cells when compared to controls [51]. Additionally, in vitro studies have reported that leptin stimulates the production of degradative enzymes, including matrix metalloproteinases (MMPs) and endoproteases [41]. Radiological and histological analyses corroborate these findings, with decreased disc height index (DHI) and increased overall degradation grades evident on histological scoring systems [41, 42]. These negative effects are not limited to NP and AF cells, with similar findings reported in studies pertaining to CEP cells [43]. Concerning lesser understood or more novel adipokines, NP and AF cells isolated from IVDs of six patients showed increased relative fold mRNA expression of pro‐inflammatory cytokines and degradative enzymes when cultured with recombinant resistin (100 ng/mL). Significant upregulation was evident for interleukin (IL)‐1β (2.68 fold), IL‐6 (10.09 fold), IL‐8 (2.52 fold), MMP‐1 (3.10 fold), MMP‐3 (2.20 fold), and MMP‐13 (2.78 fold) [44]. Significant upregulation was also observed in AF cells for the aforementioned parameters, with the greatest fold increase seen in IL‐8 (9.75 fold) and MMP‐13 (7.60 fold) [44]. Other studies have also highlighted resistin's ability to upregulate ADAMTS5 [45]. Shin et al. demonstrated that resistin exerted its effects on NP and AF cells through the NF‐Kβ pathway, as blocking this pathway with a specific inhibitor reduced resistin's impact on cellular pathological processes [25, 44].

In a study of ten patients by Hu et al. [46], the role of chemerin in DDD was highlighted. Notably, the cohort's median age, based on degenerative disc changes assessed by the Pfirrmann grade, was 28 years (range 18–46 years). Quantification of chemerin levels showed increased levels of chemerin per grade of degeneration, with a greater degree of chemerin evident in NP tissue compared to AF tissue. Furthermore, exposure of NP cells to increased levels of chemerin (1–5000 ng/mL) in vitro led to a dose‐dependent decrease in cell viability, aggrecan, SOX‐9, and type 2 collagen. Additionally, respective increases were observed in inducible nitric oxide synthase (iNOS) deficiency, COX‐2, IL‐1β, IL‐6, MMP‐13, ADAMTS5, and TNF‐α when compared to controls, indicating an inflammatory cascade potentiating the generation of potential reactive oxygen species (ROS) and ultimately cell death in young patients, leading to DDD. Similar to resistin, Hu et al. noted that chemerin mediated its effects through the NF‐Kβ pathway [46].

In contrast, certain adipokines (adiponectin, omentin‐1, progranulin) have been shown to be protective towards disc tissue. Adiponectin has been shown to downregulate inflammatory cytokines such as TNF‐α in a dose dependent (0.2‐5 μg/mL) manner, with reciprocal increases in DHI and decreases in histological degradation evident when compared to controls (IL‐β treated) [47, 48]. Comparatively, Huang et al. [49] demonstrate a dose dependent protective effect of omentin‐1 as protein expression of omentin‐1 was upregulated in NP cells cultured with increasing doses of IL‐1β (10, 20, 50 ng/mL). Analysis of senescence in cultured NP cells, per beta‐galactosidase (SA‐β‐Gal) staining, showed a stepwise reduction in overall percentage of positively stained cells for 150 and 300 ng/mL concentrations of omentin‐1 (and IL‐β: 10 ng/mL) compared to IL‐β alone. Furthermore, omentin‐1 demonstrated a dose dependent reduction in MMP‐13 and ADAMTS5 and an increase in collagen II and aggrecan when compared to IL‐β alone, thus promoting extracellular matrix synthesis in NP cells.

Other studies also demonstrate protective effects of progranulin (PGRN) in relation to IVD tissue. Wang et al. [50] observed increased levels of PGRN in degenerated discs compared to controls, associated with an increase in IL‐10. Interestingly, a positive correlation was seen between increasing levels of PGRN and visual analogue scale (VAS) scores, a commonly employed metric of patient‐reported outcome measures. In an in vivo murine model, PGRN deficiency was shown to accelerate disc degeneration, with decreased expression of IL‐10 and increased expression of IL‐17, confirmed using in vitro studies. Comparable results have been reported by Zhao et al. [51], who also reported that PGRN knockout mice exhibited enhanced activation of the NF‐Kβ pathway, resulting in degenerative processes. As observed with resistin and chemerin, which mediate their effects through this pathway, Zhao et al.'s findings suggest a potential proportional relationship between specific active adipokines and their effects, rather than the dominance of a single adipokine in DDD.

## 4 Adipokine Effects on Paraspinal Musculature

**FIGURE 3:**
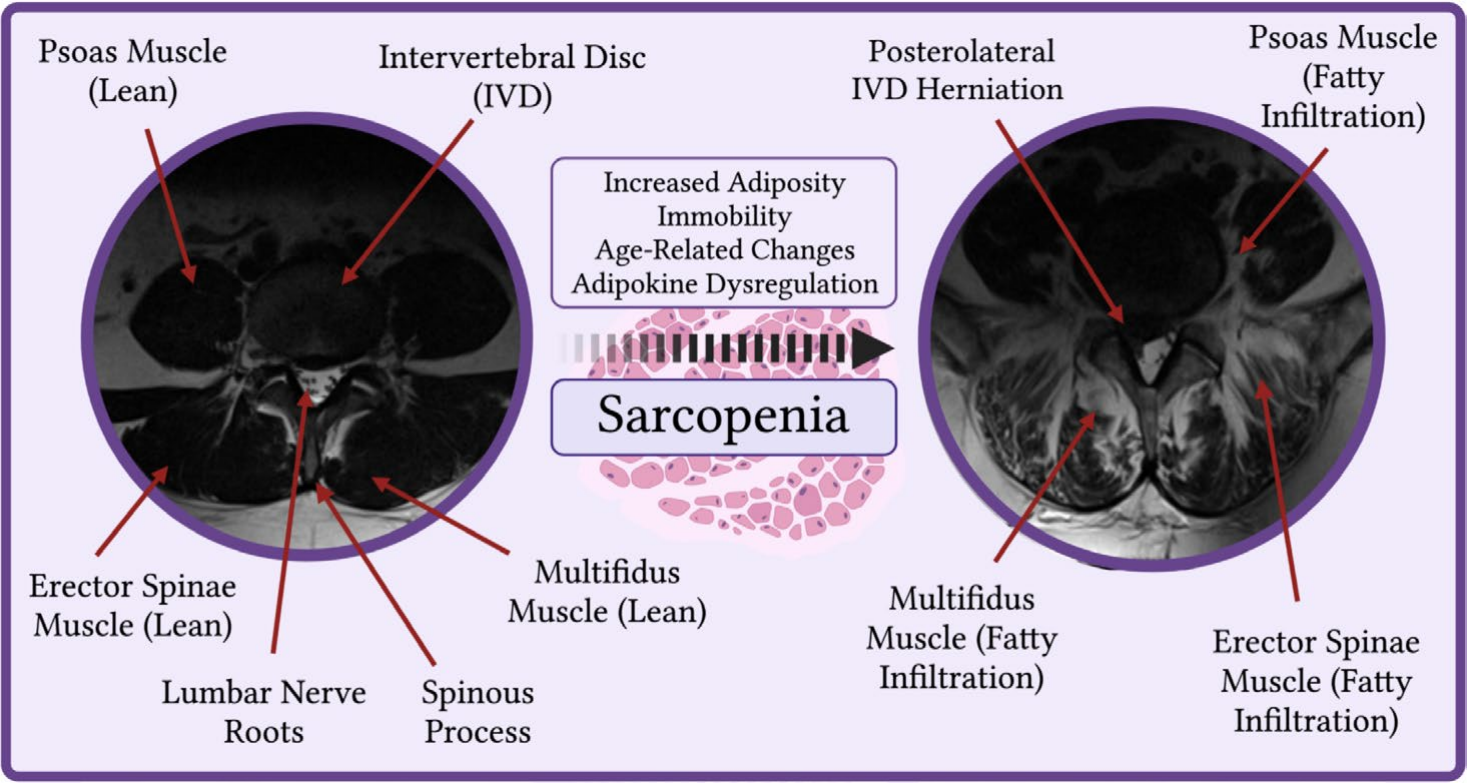
Axial T2‐weighted slice of L3‐4 spinal level on magnetic resonance imaging (MRI) highlighting comparative lean and sarcopenic paraspinal muscles (psoas, multifidus, erector spinae) in two patients, in addition to a right‐sided posterolateral herniation with compression of adjacent lumbar nerve roots.

Recently, there has been considerable interest in the realm of sarcopenia related research, defined as the loss of muscle mass and strength [52]. Histological examination of sarcopenic muscle exhibits a reduction in the number and size of muscle cells and an apparent infiltration of fibrosis and fat. It is believed that on average 5%–13% of people who are 60–70 years old have sarcopenia [52], which is associated with higher rates of postoperative complications, falls, fractures, hospital readmissions, and mortality [52, 53]. Traditionally, it is diagnosed using a clinical qualitative grading system such as the SARC‐F questionnaire. Novel measures of diagnosing sarcopenia include the use of computed tomography (CT) and magnetic resonance imaging (MRI) by measuring cross‐sectional area (CSA) and/or fat infiltration of paraspinal musculature [50]. “Central Sarcopenia” is defined as decreased CSA or increased percentage of fat infiltration in paraspinal musculature [55], typically the psoas, multifidus, and erector spinae muscle groups, as evident in Figure 3.

Certain studies have shown strong preliminary evidence regarding the influence of adipokines on the overall function of skeletal muscles. Both adiponectin and visfatin have been shown to positively promote myogenesis [56, 57, 58]. On the other hand, Wen et al. [59] reported that increased resistin levels and activity can result in ectopic lipid deposition in skeletal muscles, in addition to inhibiting adiponectin's anti‐inflammatory mechanisms. The work of Sheng et al. [53] support the findings of resistin‐mediated impairment on myogenesis by depicting reduced myotubular diameter with increased resistin levels. O'Leary et al. [61] also reported that resistin exerted a strong influence on myogenesis. Myoblasts were stimulated with resistin (5 ng/mL) for a period of 8 days, after which immunofluorescence analysis of desmin, an integral myofibrillar protein in skeletal muscle, was performed. Resistin‐treated cultures exhibited significantly reduced myotubule thickness compared to controls (*p* < 0.05). It was also shown that this effect is mediated by activation of the NF‐Kβ pathway. Comparably, available evidence for leptin is contrasting. It has been reported to activate irisin‐induced myogenesis in a murine model by Rodríguez et al. [62], while Pijet et al. [63] showed leptin impairs myogenesis in a murine cell line.

Nevertheless, adipokines appear to play an important role in myogenesis, particularly of paraspinal musculature. In a small cohort of 20 geriatric male patients admitted for elective spine surgery, Zoico et al. [64] reported a negative correlation between adiponectin, IL‐6, and peroxisome proliferator‐activated receptor gamma (PPARγ) gene expression in subcutaneous adipose tissue. PPARγ is a key factor in fatty acid oxidation and points towards an influence of oxidative stress and free radicals in the pathophysiology of this cohort [65]. Thus, exposing adipokine‐induced ROS in paraspinal musculature would prove an important finding. Similar to previous evidence by Zhang et al. [13] who noted differences in adipoR1 levels between concave and convex sides of scoliotic curves in AIS patients, Jiang et al. [66] also reported that adiponectin mRNA levels in paraspinal musculature are higher in paraspinal musculature on the concave side of the curve. Furthermore, adiponectin levels were significantly associated with the degree of the curve, as defined by the Cobb angle, possibly highlighting a paracrine effect of adipokines which can influence both neighboring skeletal muscle and bone. This was further alluded to by James et al. [67] who performed gene expression analyses of adipokines and inflammatory cytokines from muscle and fat biopsies in a cohort undergoing spine surgery for IVD herniation. The study noted a significant upfold of leptin in intramuscular and subcutaneous fat, and adiponectin in epidural fat of multifidus muscles. The authors concluded that the results of their study provide evidence of a relationship between the inflammatory state of paraspinal muscles and herniation of IVDs. Clinical studies corroborate these findings, showing significantly increased fat infiltration of local muscles on the same side of herniation [68, 69]. Nevertheless, the role of adipokines in paraspinal musculature and an overarching effect on neighboring bone and disc tissue remains in its infancy, and further research is encouraged.

## 5 Adipokine Related Pathological Inflammation of Ligaments

Ligamentous pathology was once primarily considered a degenerative disease of fibroblasts, or fibroblast‐like cells (tenocytes), resulting in disruption of the typical structure of parallel collagen fibril chains that make up ligaments and tendons [70]. However, evidence is building regarding the role of pathological inflammation induced by cytokines, exempt from age‐related degeneration, in tendon and ligament related injuries [71, 72, 73, 74, 75, 76]. While an acute inflammatory response is a crucial component of normal ligamentous healing following injury, evidence suggests that dysregulated or chronic inflammation—characterized by sustained cytokine and adipokine release—can shift the healing response towards pathological outcomes. This includes excessive fibrosis, matrix degradation, and ectopic ossification, distinct from the normal resolution and tissue repair typically expected after acute injury [71, 72, 73, 74]. Certain clinical studies have shown upregulation of inflammatory cytokines (TNF‐α, IL‐6, IL‐15, IL‐18) in supraspinatus and subscapularis tendons harvested from patients with rotator cuff tears [71, 72, 73]. Increased levels of TNF‐α have also been reported in studies concerning Achilles tendon injuries, with increased mRNA expression of cyclooxygenase‐2 (COX‐2) and IL‐6 in ruptured Achilles tendon patients compared to controls [74, 75]. In relation to adipokines in particular, higher serum levels of leptin have been shown to be indicative of lower rates of recovery from upper‐limb related connective tissue injuries, including tenosynovitis, rotator cuff tendinitis, and humeral epicondylitis, among others [76].

Regarding spinal pathology, several studies have reported preliminary findings depicting a relationship between adipokines and ossification of the posterior longitudinal ligament (OPLL) [77, 78, 79, 80, 81]. The PLL is an integral ligament to the overall stability of the spinal column and runs the length of the spine along the posterior aspect of the vertebral body [77]. OPLL is a pathological process which results in ectopic calcification of the PLL. Globally it is rare, with a worldwide prevalence of approximately 0.01%–2%. However, studies indicate a higher prevalence in Japanese populations, with the cervical spine being the most commonly affected region, with patients often presenting with radiculopathy or myelopathy [78]. Recent studies investigating the relationship between adipokines and OPLL highlight a particular influence of leptin in the pathophysiology of OPLL. Shirakura et al. [79] report higher serum leptin levels in female OPLL patients compared to controls, while no significant relationship existed for male patients.

Feng et al. [80] further emphasized leptin's ossification effect on OPLL cells in a cervical cohort of 64 patients. Although leptin had no effect on the proliferation of OPLL cells, RT‐PCR analysis depicted that leptin treated cells (50, 100, 200 ng/mL) all had significantly elevated levels of alkaline phosphatase (ALP) and osteocalcin (OCN) when compared to controls. This was shown to be mediated via phosphorylation of extracellular signal‐regulated kinase (ERK)1/2, p38 mitogen‐activated protein kinase (MAPK) and c‐Jun N‐terminal kinase (JNK) pathways. Ikeda et al. [81] noted a similar relationship. One particularly interesting finding from this study discussed a proportional relationship between leptin levels (normalized per body mass index, BMI) and the number of vertebrae involved in female patients. As previously noted in vertebral and disc related disease, sex‐specific differences may also potentially exist in OPLL cohorts.

Diffuse idiopathic skeletal hyperostosis (DISH) is a more diffuse form of spinal enthesopathy and can be associated with OPLL. Similar to OPLL, studies postulate that adipokines play a role in the pathophysiology of DISH. Tenti et al. [82] reported higher overall mean leptin and visfatin in DISH patients when compared to lumbar OA patients. While increased levels of adiponectin were evident, this did not reach statistical significance. Comparatively, Mader et al. [83] noted a significant relationship between serum adiponectin levels and syndesmophyte formation, with increased levels of adiponectin related to the extent of novel ectopic calcification.

**FIGURE 4:**
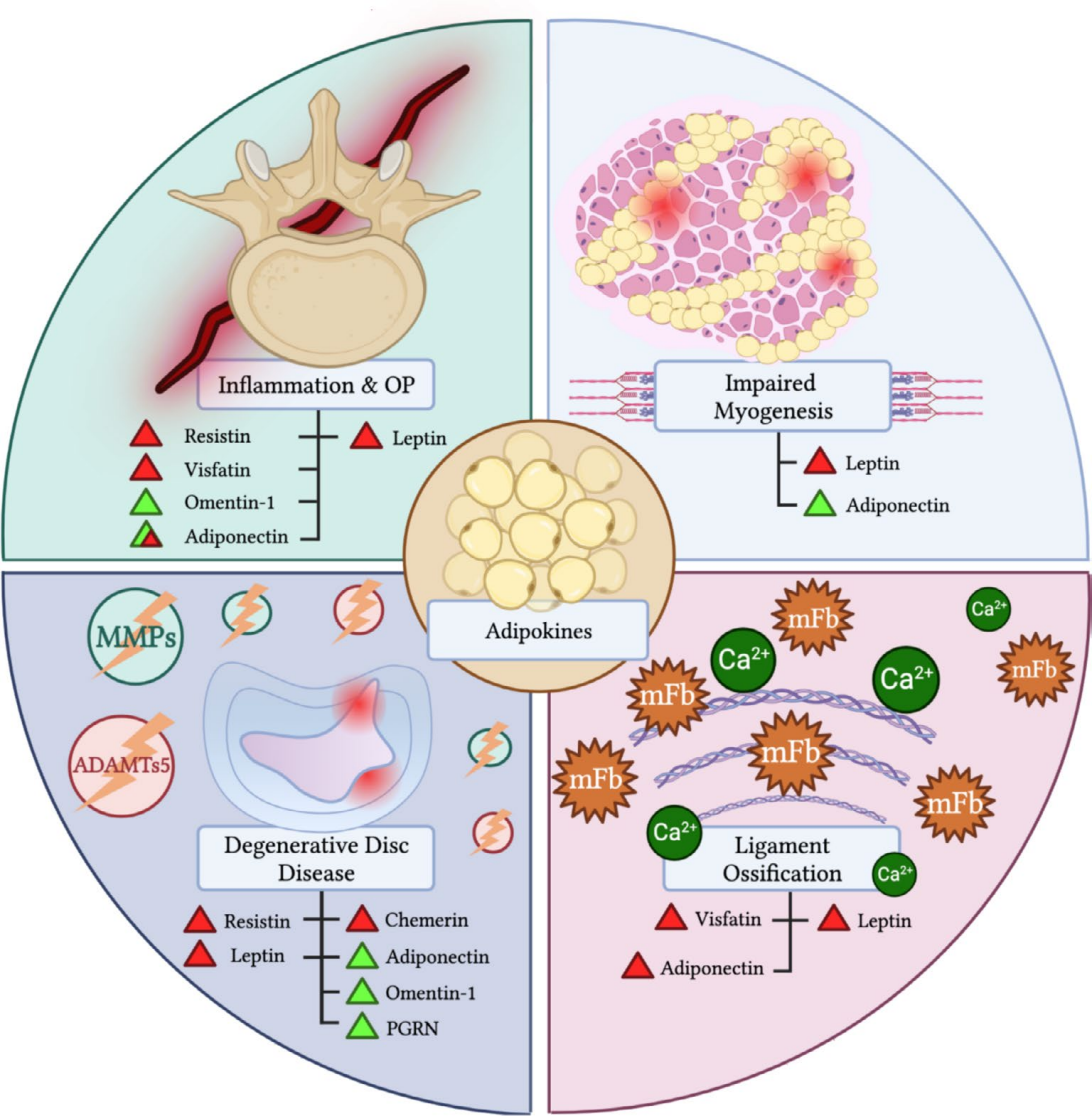
Summary of study findings concerning adipokine function in vertebral pathology, sarcopenia, degenerative disc disease and ligament ossification. (OP) = osteoporosis. (MMP) = matrix‐metalloproteinases. (ADAMTs5) = a disintegrin and metalloproteinase with thrombospondin motifs 5. (mFb) = mineralized fibroblast. (Ca^2+^) = calcium. (Green Triangle) = positive/anti‐inflammatory role. (Red Triangle) = negative/inflammatory role. (Green‐Red Triangle) = Conflicting results reported in the literature regarding inflammatory/anti‐inflammatory role.

While several clinical studies depict a causal relationship between certain adipokines and the pathology of spinal ligaments, a paucity of in vitro evidence exists. However, extra‐spinal pre‐clinical studies provide more insight into this relationship. Anti‐fibrogenic attributes of adiponectin have been highlighted in scleroderma cohorts and can occur at relevant physiological levels [84, 85, 86]. This is achieved via downregulation of signals responsible for elevated collagen gene expression. However, studies comparing Achilles rupture patients against controls found no difference in adiponectin mRNA levels between groups. Saengsoi et al. [87] highlighted using a canine model of ruptured cranial cruciate ligament that leptin negatively correlated with aggrecan levels, while visfatin was positively correlated with MMP‐13. Nevertheless, few in vitro studies exist pertaining to the effects of adipokines on fibroblasts and tenocytes isolated from spinal patients, and further studies are warranted to better delineate pathological processes. An overview of the role of adipokines identified in certain spinal pathologies is outlined in Figure 4.

## 6 Spinal Cord Injury Induced Adipokine Dysregulation

While adipokine dysregulation can precede spinal pathology related to vertebrae, IVD, muscle, and ligaments, spinal cord injury (SCI) can result in adipokine dysregulation [88]. Despite this, the association between SCI and adipokines is worth an honorable mention due to the potential for SCI‐induced adipokine dysregulation. SCI ultimately leads to neuronal damage, cell death, and can result in permanent neurological deficits. It is understandably associated with a high prevalence of injury‐associated morbidity and mortality [89]. The life expectancy of patients with SCI has increased dramatically over recent decades due to improved treatment strategies [90]. As a result of this, there have also been recent developments in understanding the physiological sequalae of living with chronic SCI. For example, neurogenic obesity secondary to SCI has been reported [91, 92]. There is increasing evidence that certain adipokines (e.g., leptin, resistin) are expressed in various regions of the central nervous system (CNS) and peripheral nervous system (PNS) [93]. Pre‐clinical models have demonstrated that induced CNS injury can result in increased mRNA expression of adipokines [94, 95]. A meta‐analysis by Latifi et al. [96] notes that plasma leptin levels are significantly higher in patients with SCI compared to controls and that the level of SCI can ultimately influence leptin levels. This can lead to prolonged activation of leptin receptors in the CNS and PNS, resulting in leptin resistance and metabolic dysfunction [97].

Adipokine dysregulation can understandably result in previously mentioned spinal pathology. For example, Doherty et al. [98] noted increased adiponectin levels in patients with SCI confined to wheelchairs when compared to those who retained the ability to walk. Tan et al. [99] demonstrated an association between adiponectin and osteoporotic fractures suffered by patients with SCI. This supports previously discussed articles highlighting an inverse relationship between adiponectin and BMD [33]. The authors reported that on univariate analysis, adiponectin levels were significantly related to femur axial stiffness and maximal load and that circulatory reduced levels of adiponectin proved a significant risk factor for the occurrence of post‐SCI osteoporotic fractures (OPFx) [99]. Furthermore, abnormal bone metabolism following SCI has been reported in studies, with heterotopic ossification evident in multiple joints following CNS trauma [100]. While SCI has also been associated with decreased muscle mass and increased fat infiltration of skeletal muscle, this is believed to be predominantly related to immobilization and decreased physical activity post‐injury rather than adipokine dysregulation. Similarly, there is a lack of robust evidence pertaining to degenerative disc and ligamentous changes following SCI. Nevertheless, adipokine dysregulation secondary to SCI could provide an important avenue for novel therapeutic strategies.

## 7 Managing Circulating Pro‐Inflammatory Adipokine Levels With Natural and Synthetic Compounds

A multitude of naturally occurring and synthetic compounds can positively influence circulatory adipokine levels and overall physiological function. It is well established that several diabetic medications can reduce systemic adipokine levels. Common medications include metformin and various formulations of glitazones and glucose‐like peptide‐1 (GLP‐1) receptor agonists [101, 102, 103, 104, 105, 106, 107]. However, their role in spinal disease is less established. The majority of preliminary evidence relates to the ability of certain prescription medications to suppress pathological processes related to IVD degeneration. For example, Chen et al. [104] depict that metformin can protect NP cells from tert‐Butyl hydroperoxide (TBHP) induced apoptosis in vitro in a dose‐dependent manner (10–200 μM) and can reduce the degree of disc degeneration observed in vivo at both 8‐ and 16‐weeks postoperatively. Similarly, Hu et al. [103] reported that pioglitazone can protect NP cells from mechanically induced compressive stress, reducing the degree of apoptosis evident in cultures. Yao et al. [105] reported that liraglutide, a GLP‐1 receptor agonist, can reduce NP cell apoptosis when subjected to a high glucose (0.2 M) environment for 48 h. However, further evidence regarding the role of these medications in spinal pathology is needed.

Concerning naturally occurring compounds, several studies exist highlighting the efficacy of particular substances in reducing circulatory levels of pro‐inflammatory adipokines. Graf et al. [108] reported that anthocyanin, a pigment which accumulates in the vacuoles of plants, can reduce systemic levels of leptin and resistin in a rodent model. Anthocyanins are classified as flavonoids, which represent one of three major pigments found in plant species. The other two are chlorophylls and carotenoids [108, 109, 110]. Ten weeks of treatment with a high‐anthocyanin diet led to significant reductions in serum leptin and resistin when compared to controls. However, this was not seen in mesenteric adipose tissue (MAT) analysis, a site of adipose tissue in rodents believed to closely resemble that of visceral adipose tissue in humans [109]. Similarly, no differences were evident for serum and MAT adiponectin levels.

The ability to reduce inflammation has also been noted for carotenoids. Lycopene supplementation for six weeks has been shown to reduce levels of leptin, resistin, IL‐6, and TNF‐α when compared to controls in a rodent model [109]. Similar findings have been reported for β‐carotene, which can reportedly regulate the NF‐Kβ pathway, a pivotal pathway in certain adipokine‐mediated pathological processes discussed throughout this article [110].

Other natural substances such as diosgenin (DG), classified as a phytosteroid sapogenin, depict similar benefits [111, 112, 113]. Khateeb et al. [111] compared normal diet (ND) versus a high fat diet (HFD) +/− diosgenin (NFD + DG) in a murine model. Akin to flavonoids and carotenoids, 6 weeks of treatment with DG was shown to reduce IL‐6 levels compared to HFD, with an overall 45% reduction in IL‐6 levels reported. Chen et al. [112] further support these findings, reporting that DG can decrease pro‐inflammatory adipokine levels via regulation of the AMP‐activated protein kinase (AMPK) pathway. Additionally, polyphenols such as resveratrol can also downregulate pro‐inflammatory adipokines [113].

In terms of anti‐inflammatory adipokines, Landrier et al. [114] reported that treatment with certain polyphenols such as y‐tocopherol (vitamin E) resulted in increased plasma adiponectin levels when compared to controls. This was confirmed in vitro with 3T3‐L1 preadipocytes, as treatment with both α‐tocopherol and γ‐tocopherol led to a dose‐dependent (10, 25, 50 μM) increase in adiponectin mRNA levels through a PPARγ dependent mechanism. In a similar study by Lira et al. [115], supplementation of both α‐tocopherol (50 μM) and vitamin D3 (0.1 μM) for 24 h led to decreased levels of lipopolysaccharide (LPS) induced IL‐6. An overall 88% reduction was evident in cells treated with α‐tocopherol, while a 60% overall reduction was observed in cells treated with vitamin D3. The capacity to upregulate anti‐inflammatory adipokines, as demonstrated in studies relating to polyphenols, has also been noted in the literature for phytosteroid compounds such as genistein [116].

**FIGURE 5:**
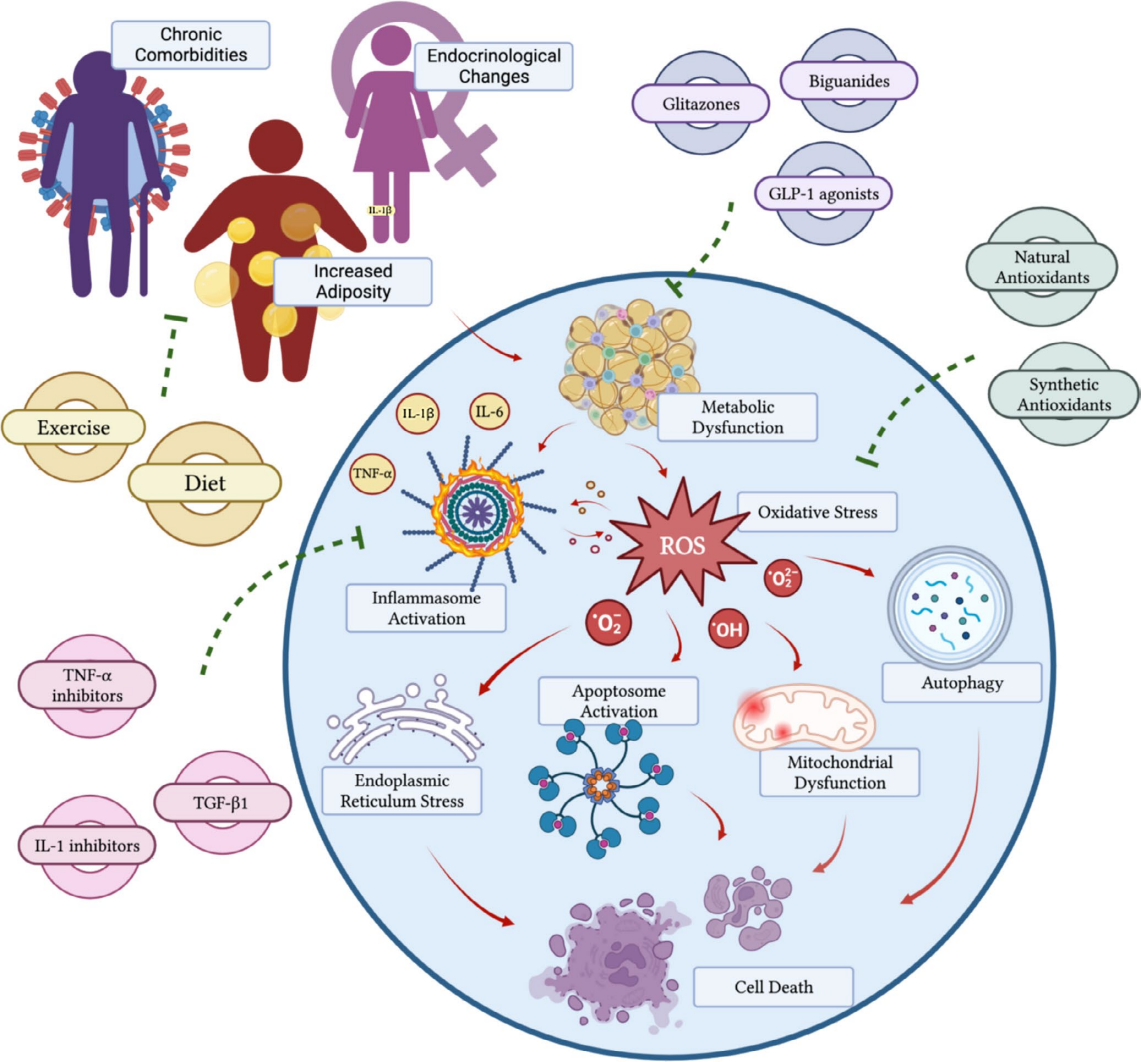
Novel therapeutic options for adipokine dysregulation and consequential sequelae. (TNF‐α) = tumor necrosis factor‐alpha. (TGF‐β1) = transforming growth factor‐beta 1. (IL‐1) = interleukin‐1. (IL‐1β). = interleukin‐1β. (IL‐6) = interleukin‐6. (GLP‐1) = glucagon like peptide‐1. (ROS) = reactive oxygen species. (O2^−^) = superoxide free radical. (O2^−2^) = peroxide free radical. (OH^−^) = hydroxide free radical.

The aforementioned compounds are naturally occurring and commonly found in plants, fruits, vegetables, nuts, and dairy products. All possess potent antioxidant properties, alluding to a major link between adipokine levels, oxidative stress, and pathological processes, as previously discussed throughout this article [65, 103, 111, 117]. Furthermore, it poses the question: could dietary intervention benefit patients presenting with spinal disease if believed to be mediated by increased systemic levels of pro‐inflammatory adipokines and ROS generation. In a randomized controlled trial (RCT) by Lederer et al. [118], the authors evaluated the effect of a 4‐week vegan diet on plasma adiponectin and leptin levels. A decrease in leptin levels was evident in both vegan and meat‐rich diets, which did not prove significant. However, there was a statistically significant difference evident in adiponectin levels. For the meat‐rich diet cohort, adiponectin decreased from baseline to end measurement. Comparatively, adiponectin levels increased in the vegan diet cohort. Thus, the results of this study provide preliminary evidence to support the anti‐inflammatory properties of a vegan diet. However, other studies regarding vegetarian diets have shown conflicting results. Ambroszkiewicz et al. [119] note in their study that a vegetarian diet only positively affected serum levels of leptin and did not affect levels of adiponectin, visfatin, and omentin. It is important to note that their study was cross‐sectional in design, concerned prepubertal children, and self‐reported dietary status. Age and sex of cohorts may be important considerations, as both patient‐related factors may influence the overall effect particular diets have on adipokine levels [120], a common theme noted in this article. Nevertheless, as discussed above and outlined in Figure 5, several therapeutic options exist to address adipokine dysregulation and ROS generation. However, their efficacy in treating clinical spinal disease has not been fully elucidated. Further studies are needed to investigate the role of diet, natural substances, and exogenous prescription medications in regulating adipokine levels and ROS species in specific pathological states relating to spinal disease.

## 8 Conclusions and Future Outlook

Hundreds of adipokines have been identified, though the precise functions of many remain unclear. While adipokines play a crucial role in maintaining normal physiological processes, disruptions in their homeostatic levels can lead to pathological outcomes. Adiponectin and leptin are the most abundant adipokines secreted by adipose tissue. Adiponectin is primarily associated with anti‐inflammatory properties, while leptin has been shown to promote a self‐preserved cycle of inflammation. Adipokine dysregulation, characterized by an imbalance that promotes inflammation, has been implicated in various disease states. This imbalance can result in catastrophic cellular sequelae, such as the generation of ROS, endoplasmic reticulum stress, mitochondrial dysfunction, autophagy, and activation of apoptosomes, ultimately leading to cell death.

Concerning spinal disease, this article outlines the interplay of adipokines in inflammatory states. In bone, this can lead to bone resorption and reduced bone mineral density. In intervertebral disc tissue, this can result in degenerative disc disease. While in paraspinal musculature and ligaments, adipokine imbalance can influence sarcopenic changes and ossification, respectively. SCI can lead to dysregulation of adipokines, characterized by increased levels of leptin in affected individuals, highlighting the complex interplay between neurological injury and adipokine signaling. Favorably, numerous therapeutic strategies exist that can interrupt the aforementioned mechanisms of cellular demise. These approaches include conservative measures such as diet and exercise, along with commonly prescribed medications possessing inherent antioxidant properties. While in vitro results are promising, their effectiveness in clinical spinal populations remains uncertain. Therefore, further robust pre‐clinical and clinical studies are needed to clarify the precise role of adipokines in spinal diseases, their associated pathophysiology, and the efficacy of novel treatment strategies.

## Author Contributions

All authors significantly contributed to the article's conception and design. Jake M. McDonnell was responsible for interpreting the literature data and drafting the article. Stacey L. Darwish and Joseph S. Butler contributed to the interpretation of literature data. As the overall project funding holder, Conor T. Buckley ensured the work's integrity from inception to finalization and provided substantial contributions to the interpretation of literature. All authors critically reviewed the work prior to submission and approved the final submitted manuscript.

## Conflicts of Interest

Conor T. Buckley is an Editorial Board member of JOR Spine and co‐author of this article. They were excluded from editorial decision‐making related to the acceptance of this article for publication in the journal.

## References

[1] N. Ouchi , J. Parker , J. Lugus , and K. Walsh , “Adipokines in Inflammation and Metabolic Disease,” Nature Reviews Immunology 11 (2011): 85–97. 10.1038/nri2921 PMC3518031 21252989

[2] S. H. Jeet , “The Unfolding Tale of Leptin,” Malayssian Journal of Medical Sciences 8, no. 2 (2001): 1–6. PMC3413641 22893752

[3] S. Lehr , S. Hartwig , and H. Sell , “Adipokines: A Treasure Trove for the Discovery of Biomarkers for Metabolic Disorders,” Proteomics. Clinical Applications 6 (2012): 91–101. 22213627 10.1002/prca.201100052

[4] G. Fantuzzi , “Adipose Tissue, Adipokines, and Inflammation,” Journal of Allergy and Clinical Immunology 115, no. 5 (2005): 911–919. 15867843 10.1016/j.jaci.2005.02.023

[5] M. Blüher , “Clinical Relevance of Adipokines,” Diabetes and Metabolism Journal 36, no. 5 (2012): 317–327. 23130315 10.4093/dmj.2012.36.5.317 PMC3486977

[6] M. Fasshauer and M. Blüher , “Adipokines in Health and Disease,” Trends in Pharmacological Sciences 36, no. 7 (2015): 461–470. 26022934 10.1016/j.tips.2015.04.014

[7] P. Mancuso , “The Role of Adipokines in Chronic Inflammation,” ImmunoTargets and Therapy 5 (2016): 47–56. 27529061 10.2147/ITT.S73223 PMC4970637

[8] T. Kawai , M. V. Autieri , and R. Scalia , “Adipose Tissue Inflammation and Metabolic Dysfunction in Obesity,” American Journal of Physiology. Cell Physiology 320, no. 3 (2021): C375–C391. 33356944 10.1152/ajpcell.00379.2020 PMC8294624

[9] H. Kwon and J. E. Pessin , “Adipokines Mediate Inflammation and Insulin Resistance,” Frontiers in Endocrinology 4 (2013): 71. 23781214 10.3389/fendo.2013.00071 PMC3679475

[10] L. C. Freitas Lima , V. A. Braga , M. do Socorro de França Silva , et al., “Adipokines, Diabetes and Atherosclerosis: An Inflammatory Association,” Frontiers in Physiology 6 (2015): 304. 26578976 10.3389/fphys.2015.00304 PMC4630286

[11] C. W. Zhao , Y. H. Gao , W. X. Song , et al., “An Update on the Emerging Role of Resistin on the Pathogenesis of Osteoarthritis,” Mediators of Inflammation 2019 (2019): 1–8. 1532164. 10.1155/2019/1532164 PMC6369476 30809105

[12] A. Hartl , J. Sieper , U. Syrbe , et al., “Serum Levels of Leptin and High Molecular Weight Adiponectin Are Inversely Associated With Radiographic Spinal Progression in Patients With Ankylosing Spondylitis: Results From the ENRADAS Trial,” Arthritis Research & Therapy 19, no. 1 (2017): 140. 28619118 10.1186/s13075-017-1350-9 PMC5471667

[13] H. Q. Zhang , L. J. Wang , S. H. Liu , J. Li , L. G. Xiao , and G. T. Yang , “Adiponectin Regulates Bone Mass in AIS Osteopenia via RANKL/OPG and IL6 Pathway,” Journal of Translational Medicine 17, no. 1 (2019): 64. 30819183 10.1186/s12967-019-1805-7 PMC6396498

[14] V. Francisco , J. Pino , M. Á. González‐Gay , et al., “A New Immunometabolic Perspective of Intervertebral Disc Degeneration,” Nature Reviews Rheumatology 18, no. 1 (2022): 47–60. 34845360 10.1038/s41584-021-00713-z

[15] V. Francisco , D. Ait Eldjoudi , M. González‐Rodríguez , et al., “Metabolomic Signature and Molecular Profile of Normal and Degenerated Human Intervertebral Disc Cells,” Spine Journal 23, no. 10 (2023): 1549–1562. 10.1016/j.spinee.2023.06.005 37339697

[16] C. Ruiz‐Fernández , V. Francisco , J. Pino , et al., “Molecular Relationships Among Obesity, Inflammation and Intervertebral Disc Degeneration: Are Adipokines the Common Link?,” International Journal of Molecular Sciences 20, no. 8 (2019): 2030. 31027158 10.3390/ijms20082030 PMC6515363

[17] M. González‐Rodríguez , D. Ait Edjoudi , A. Cordero Barreal , et al., “Progranulin in Musculoskeletal Inflammatory and Degenerative Disorders, Focus on Rheumatoid Arthritis, Lupus and Intervertebral Disc Disease: A Systematic Review,” Pharmaceuticals (Basel) 15, no. 12 (2022): 1544. 36558994 10.3390/ph15121544 PMC9782117

[18] Y. Farrag , M. Farrag , M. Varela‐García , et al., “Adipokines as Potential Pharmacological Targets for Immune Inflammatory Rheumatic Diseases: Focus on Rheumatoid Arthritis, Osteoarthritis, and Intervertebral Disc Degeneration,” Pharmacological Research 205 (2024): 107219. 38763327 10.1016/j.phrs.2024.107219

[19] M. González‐Rodríguez , D. Ait Eldjoudi , A. Cordero‐Barreal , et al., “E74‐Like ETS Transcription Factor 3 Expression and Regulation in Human Intervertebral Disc,” JOR Spine 8, no. 1 (2025): e70016. 39877798 10.1002/jsp2.70016 PMC11774240

[20] C. Ruiz‐Fernández , D. Ait Eldjoudi , M. González‐Rodríguez , et al., “Monomeric CRP Regulates Inflammatory Responses in Human Intervertebral Disc Cells,” Bone & Joint Research 12, no. 3 (2023): 189–198. 37051830 10.1302/2046-3758.123.BJR-2022-0223.R1 PMC10032231

[21] L. Thommesen , A. K. Stunes , M. Monjo , et al., “Expression and Regulation of Resistin in Osteoblasts and Osteoclasts Indicate a Role in Bone Metabolism,” Journal of Cellular Biochemistry 99, no. 3 (2006): 824–834. 16721825 10.1002/jcb.20915

[22] S. Zheng , J. Xu , S. Xu , et al., “Association Between Circulating Adipokines, Radiographic Changes, and Knee Cartilage Volume in Patients With Knee Osteoarthritis,” Scandinavian Journal of Rheumatology 45, no. 3 (2016): 224–229. 26505548 10.3109/03009742.2015.1083053

[23] S. Tariq , S. Tariq , S. Khaliq , and K. P. Lone , “Serum Resistin Levels and Related Genetic Variants Are Associated With Bone Mineral Density in Postmenopausal Women,” Frontiers in Endocrinology 13 (2022): 868120. 35992125 10.3389/fendo.2022.868120 PMC9389046

[24] J. H. Lee , T. Ort , K. Ma , et al., “Resistin Is Elevated Following Traumatic Joint Injury and Causes Matrix Degradation and Release of Inflammatory Cytokines From Articular Cartilage In Vitro,” Osteoarthritis and Cartilage 17, no. 5 (2009): 613–620. 19095472 10.1016/j.joca.2008.08.007

[25] J. Shin , S. Park , H. Cho , J. H. Kim , and H. Choi , “Adipokine Human Resistin Promotes Obesity‐Associated Inflammatory Intervertebral Disc Degeneration via Pro‐Inflammatory Cytokine Cascade Activation,” Scientific Reports 12 (2022): 8936. 35624126 10.1038/s41598-022-12793-2 PMC9142523

[26] A. M. Philp , R. L. Collier , L. M. Grover , E. T. Davis , and S. W. Jones , “Resistin Promotes the Abnormal Type I Collagen Phenotype of Subchondral Bone in Obese Patients With End Stage Hip Osteoarthritis,” Scientific Reports 7 (2017): 4042. 28642544 10.1038/s41598-017-04119-4 PMC5481425

[27] E. Toussirot , F. Michel , M. Béreau , et al., “Serum Adipokines, Adipose Tissue Measurements and Metabolic Parameters in Patients With Advanced Radiographic Knee Osteoarthritis,” Clinical Rheumatology 36, no. 11 (2017): 2531–2539. 28831581 10.1007/s10067-017-3789-0

[28] X. Ma , X. Zhu , X. He , X. Yi , and A. Jin , “The Wnt Pathway Regulator Expression Levels and Their Relationship to Bone Metabolism in Thoracolumbar Osteoporotic Vertebral Compression Fracture Patients,” American Journal of Translational Research 13, no. 5 (2021): 4812–4818. 34150062 PMC8205725

[29] E. Franco‐Trepat , M. Guillán‐Fresco , A. Alonso‐Pérez , et al., “Visfatin Connection: Present and Future in Osteoarthritis and Osteoporosis,” Journal of Clinical Medicine 8, no. 8 (2019): 1178. 31394795 10.3390/jcm8081178 PMC6723538

[30] J. Ma , D. S. Niu , N. J. Wan , Y. Qin , and C. J. Guo , “Elevated Chemerin Levels in Synovial Fluid and Synovial Membrane From Patients With Knee Osteoarthritis,” International Journal of Clinical and Experimental Pathology 8, no. 10 (2015): 13393–13398. 26722546 PMC4680491

[31] S. S. Rao , Y. Hu , P. L. Xie , et al., “Omentin‐1 Prevents Inflammation‐Induced Osteoporosis by Downregulating the Pro‐Inflammatory Cytokines,” Bone Research 30, no. 6 (2018): 9. 10.1038/s41413-018-0012-0 PMC5876344 29619269

[32] M. Yamauchi , T. Sugimoto , T. Yamaguchi , et al., “Plasma Leptin Concentrations Are Associated With Bone Mineral Density and the Presence of Vertebral Fractures in Postmenopausal Women,” Clinical Endocrinology 55, no. 3 (2001): 341–347. 11589677 10.1046/j.1365-2265.2001.01361.x

[33] M. R. Araneta , D. von Mühlen , and E. Barrett‐Connor , “Sex Differences in the Association Between Adiponectin and BMD, Bone Loss, and Fractures: The Rancho Bernardo Study,” Journal of Bone and Mineral Research 24, no. 12 (2009): 2016–2022. 19453256 10.1359/JBMR.090519 PMC2791515

[34] U. Syrbe , J. Callhoff , K. Conrad , et al., “Serum Adipokine Levels in Patients With Ankylosing Spondylitis and Their Relationship to Clinical Parameters and Radiographic Spinal Progression,” Arthritis & Rhematology 67, no. 3 (2015): 678–685. 10.1002/art.38968 25417763

[35] J. Rademacher , M. Siderius , L. Gellert , et al., “Baseline Serum Biomarkers of Inflammation, Bone Turnover and Adipokines Predict Spinal Radiographic Progression in Ankylosing Spondylitis Patients on TNF Inhibitor Therapy,” Seminars in Arthritis and Rheumatism 53 (2022): 151974. 35150984 10.1016/j.semarthrit.2022.151974

[36] J. H. Park , S. G. Lee , Y. K. Jeon , E. K. Park , Y. S. Suh , and H. O. Kim , “Relationship Between Serum Adipokine Levels and Radiographic Progression in Patients With Ankylosing Spondylitis: A Preliminary 2‐Year Longitudinal Study,” Medicine (Baltimore) 96, no. 33 (2017): e7854. 28816988 10.1097/MD.0000000000007854 PMC5571725

[37] Q. Wang , C. Wang , W. Hu , F. Hu , W. Liu , and X. Zhang , “Disordered Leptin and Ghrelin Bioactivity in Adolescent Idiopathic Scoliosis (AIS): A Systematic Review and Meta‐Analysis,” Journal of Orthopaedic Surgery and Research 15 (2020): 502. 33121521 10.1186/s13018-020-01988-w PMC7596938

[38] E. Normand , A. Franco , N. Alos , S. Parent , A. Moreau , and V. Marcil , “Circulatory Adipokines and Incretins in Adolescent Idiopathic Scoliosis: A Pilot Study,” Children (Basel) 9, no. 11 (2022): 1619. 36360347 10.3390/children9111619 PMC9688531

[39] S. Virk , A. L. Bertone , H. H. Hussein , J. M. Toth , M. Kaido , and S. Khan , “The Temporal Expression of Adipokines During Spinal Fusion,” Spine Journal 17, no. 12 (2017): 1897–1906. 10.1016/j.spinee.2017.06.019 28647583

[40] Y. S. Choi , “Pathophysiology of Degenerative Disc Disease,” Asian Spine Journal 3, no. 1 (2009): 39–44. 20404946 10.4184/asj.2009.3.1.39 PMC2852042

[41] A. H. Segar , J. C. T. Fairbank , and J. Urban , “Leptin and the Intervertebral Disc: A Biochemical Link Exists Between Obesity, Intervertebral Disc Degeneration and Low Back Pain—An In Vitro Study in a Bovine Model,” European Spine Journal 28 (2019): 214–223. 30324498 10.1007/s00586-018-5778-7

[42] A. Lai , J. Gansau , S. E. Gullbrand , et al., “Development of a Standardized Histopathology Scoring System for Intervertebral Disc Degeneration in Rat Models: An Initiative of the ORS Spine Section,” JOR Spine 4 (2021): e1150. 34337335 10.1002/jsp2.1150 PMC8313153

[43] Y. C. Han , B. Ma , S. Guo , et al., “Leptin Regulates Disc Cartilage Endplate Degeneration and Ossification Through Activation of the MAPK‐ERK Signalling Pathway In Vivo and In Vitro,” Journal of Cellular and Molecular Medicine 22, no. 4 (2018): 2098–2109. 29372627 10.1111/jcmm.13398 PMC5867127

[44] Z. Zhang , J. Du , Q. Xu , et al., “Resistin Promotes Nasopharyngeal Carcinoma Metastasis Through TLR4‐Mediated Activation of p38 MAPK/NF‐κB Signaling Pathway,” Cancers (Basel) 14, no. 23 (2022): 6003. 36497484 10.3390/cancers14236003 PMC9737889

[45] C. Liu , H. Yang , F. Gao , et al., “Resistin Promotes Intervertebral Disc Degeneration by Upregulation of ADAMTS‐5 Through p38 MAPK Signaling Pathway,” Spine (Phila Pa 1976) 41, no. 18 (2016): 1414–1420. 26974833 10.1097/BRS.0000000000001556

[46] S. Hu , Z. Shao , C. Zhang , et al., “Chemerin Facilitates Intervertebral Disc *Degeneration* via TLR4 and CMKLR1 and Activation of NF‐kB Signaling Pathway,” Aging (Albany NY) 12, no. 12 (2020): 11732–11753. 32526705 10.18632/aging.103339 PMC7343479

[47] B. Yuan , L. Huang , M. Yan , et al., “Adiponectin Downregulates TNF‐α Expression in Degenerated Intervertebral Discs,” Spine (Phila Pa 1976) 43, no. 7 (2018): E381–E389. 28767622 10.1097/BRS.0000000000002364

[48] H. Ohnishi , Z. Zhang , T. Yurube , et al., “Anti‐Inflammatory Effects of Adiponectin Receptor Agonist AdipoRon Against Intervertebral Disc Degeneration,” International Journal of Molecular Sciences 24, no. 10 (2023): 8566. 37239908 10.3390/ijms24108566 PMC10217873

[49] X. Huang , C. Chen , Y. Chen , J. Xu , and L. Liu , “Omentin‐1 Alleviate Interleukin‐1β(IL‐1β)‐induced Nucleus Pulposus Cells Senescence,” Bioengineered 13, no. 5 (2022): 13849–13859. 35707832 10.1080/21655979.2022.2084495 PMC9275897

[50] S. Wang , J. Wei , Y. Fan , et al., “Progranulin Is Positively Associated With Intervertebral Disc Degeneration by Interaction With IL‐10 and IL‐17 Through TNF Pathways,” Inflammation 41, no. 5 (2018): 1852–1863. 29992506 10.1007/s10753-018-0828-1

[51] Y. Zhao , Q. Tian , B. Liu , et al., “Progranulin Knockout Accelerates Intervertebral Disc Degeneration in Aging Mice,” Scientific Reports 5 (2015): 9102. 25777988 10.1038/srep09102 PMC4894449

[52] S. Von Haehling , J. E. Morley , and S. D. Anker , “An Overview of Sarcopenia: Facts and Numbers on Prevalence and Clinical Impact,” Journal of Cachexia, Sarcopenia and Muscle 1, no. 2 (2010): 129–133. 21475695 10.1007/s13539-010-0014-2 PMC3060646

[53] L. Z. Pipek , C. G. Baptista , R. F. V. Nascimento , et al., “The Impact of Properly Diagnosed Sarcopenia on Postoperative Outcomes After Gastrointestinal Surgery: A Systematic Review and Meta‐Analysis,” PLoS One 15, no. 8 (2020): e0237740. 32822372 10.1371/journal.pone.0237740 PMC7446889

[54] D. Gibbons , J. McDonnell , D. Ahern , et al., “The Relationship Between Radiological Paraspinal Lumbar Measures and Clinical Measures of Sarcopenia in Older Patients With Chronic Lower Back Pain,” Journal of Frailty & Sarcopenia & Falls 7, no. 2 (2022): 52–59. 10.22540/JFSF-07-052 PMC9175279 35775088

[55] D. U. Kim , H. K. Park , G. H. Lee , et al., “Central Sarcopenia, Frailty and Comorbidity as Predictor of Surgical Outcome in Elderly Patients With Degenerative Spine Disease,” Journal of Korean Neurosurgical Association 64, no. 6 (2021): 995–1003. 10.3340/jkns.2021.0074 PMC8590910 34614555

[56] E. Arabzadeh , H. Shirvani , M. Ebadi Zahmatkesh , S. Riyahi Malayeri , G. H. Meftahi , and F. Rostamkhani , “Irisin/FNDC5 Influences Myogenic Markers on Skeletal Muscle Following High and Moderate‐Intensity Exercise Training in STZ‐Diabetic Rats,” 3 Biotech 12, no. 9 (2022): 193. 10.1007/s13205-022-03253-9 PMC9325938 35910290

[57] S. M. Krzysik‐Walker , J. A. Hadley , J. E. Pesall , D. C. McFarland , R. Vasilatos‐Younken , and R. Ramachandran , “Nampt/Visfatin/PBEF Affects Expression of Myogenic Regulatory Factors and Is Regulated by Interleukin‐6 in Chicken Skeletal Muscle Cells,” Comparative Biochemistry and Physiology. Part A, Molecular & Integrative Physiology 159, no. 4 (2011): 413–421. 10.1016/j.cbpa.2011.04.007 21545843

[58] A. Yu , Y. Zheng , Y. Gong , R. Yu , Z. Yang , and X. Chen , “Adiponectin Promotes Myogenic Differentiation via a Mef2C‐AdipoR1 Positive Feedback Loop,” Gene 771 (2021): 145380. 33359123 10.1016/j.gene.2020.145380

[59] F. Wen , J. Hou , X. Ji , et al., “The Mef2c/AdipoR1 Axis Is Responsible for Myogenic Differentiation and Is Regulated by Resistin in Skeletal Muscles,” Gene 857 (2023): 147193. 36641076 10.1016/j.gene.2023.147193

[60] C. H. Sheng , Z. W. Du , Y. Song , et al., “Human Resistin Inhibits Myogenic Differentiation and Induces Insulin Resistance in Myocytes,” BioMed Research International 2013 (2013): 1–8. 804632. 10.1155/2013/804632 PMC3590612 23509781

[61] M. F. O'Leary , G. R. Wallace , E. T. Davis , et al., “Obese Subcutaneous Adipose Tissue Impairs Human Myogenesis, Particularly in Old Skeletal Muscle, via Resistin‐Mediated Activation of NFκB,” Scientific Reports 8 (2018): 15360. 30337633 10.1038/s41598-018-33840-x PMC6193975

[62] A. Rodríguez , S. Becerril , L. Méndez‐Giménez , et al., “Leptin Administration Activates Irisin‐Induced Myogenesis via Nitric Oxide‐Dependent Mechanisms, but Reduces Its Effect on Subcutaneous Fat Browning in Mice,” International Journal of Obesity 39, no. 3 (2015): 397–407. 25199621 10.1038/ijo.2014.166

[63] M. Pijet , B. Pijet , A. Litwiniuk , B. Pajak , B. Gajkowska , and A. Orzechowski , “Leptin Impairs Myogenesis in C2C12 Cells Through JAK/STAT and MEK Signaling Pathways,” Cytokine 61, no. 2 (2013): 445–454. 23201486 10.1016/j.cyto.2012.11.002

[64] E. Zoico , A. Rossi , V. Di Francesco , et al., “Adipose Tissue Infiltration in Skeletal Muscle of Healthy Elderly Men: Relationships With Body Composition, Insulin Resistance, and Inflammation at the Systemic and Tissue Level,” Journals of Gerontology. Series A, Biological Sciences and Medical Sciences 65, no. 3 (2010): 295–299. 19864639 10.1093/gerona/glp155 PMC4051307

[65] J. Li , M. Tang , G. Yang , L. Wang , Q. Gao , and H. Zhang , “Muscle Injury Associated Elevated Oxidative Stress and Abnormal Myogenesis in Patients With Idiopathic Scoliosis,” International Journal of Biological Sciences 15, no. 12 (2019): 2584–2595. 31754331 10.7150/ijbs.33340 PMC6854377

[66] H. Jiang , F. Yang , T. Lin , et al., “Asymmetric Expression of H19 and ADIPOQ in Concave/Convex Paravertebral Muscles Is Associated With Severe Adolescent Idiopathic Scoliosis,” Molecular Medicine 24, no. 1 (2018): 48. 30241458 10.1186/s10020-018-0049-y PMC6145194

[67] G. James , X. Chen , A. Diwan , and P. W. Hodges , “Fat Infiltration in the Multifidus Muscle Is Related to Inflammatory Cytokine Expression in the Muscle and Epidural Adipose Tissue in Individuals Undergoing Surgery for Intervertebral Disc Herniation,” European Spine Journal 30, no. 4 (2021): 837–845. 32594231 10.1007/s00586-020-06514-4

[68] M. C. Battié , R. Niemelainen , L. E. Gibbons , and S. Dhillon , “Is Level‐ and Side‐Specific Multifidus Asymmetry a Marker for Lumbar Disc Pathology?,” Spine Journal 12, no. 10 (2012): 932–939. 10.1016/j.spinee.2012.08.020 23084154

[69] M. Fortin , À. Lazáry , P. P. Varga , I. McCall , and M. C. Battié , “Paraspinal Muscle Asymmetry and Fat Infiltration in Patients With Symptomatic Disc Herniation,” European Spine Journal 25, no. 5 (2016): 1452–1459. 26957101 10.1007/s00586-016-4503-7

[70] C. B. Frank , “Ligament Structure, Physiology and Function,” Journal of Musculoskeletal & Neuronal Interactions 4, no. 2 (2004): 199–201. 15615126

[71] N. L. Millar , A. Q. Wei , T. J. Molloy , F. Bonar , and G. A. Murrell , “Cytokines and Apoptosis in Supraspinatus Tendinopathy,” Journal of Bone and Joint Surgery. British Volume 91, no. 3 (2009): 417–424. 19258623 10.1302/0301-620X.91B3.21652

[72] J. E. Gaida , J. Bagge , C. Purdam , J. Cook , H. Alfredson , and S. Forsgren , “Evidence of the TNF‐α System in the Human Achilles Tendon: Expression of TNF‐α and TNF Receptor at Both Protein and mRNA Levels in the Tenocytes,” Cells, Tissues, Organs 196, no. 4 (2012): 339–352. 22572155 10.1159/000335475

[73] M. K. Shindle , C. C. Chen , C. Robertson , et al., “Full‐Thickness Supraspinatus Tears Are Associated With More Synovial Inflammation and Tissue Degeneration Than Partial‐Thickness Tears,” Journal of Shoulder and Elbow Surgery 20, no. 6 (2011): 917–927. 21612944 10.1016/j.jse.2011.02.015 PMC3156316

[74] K. Legerlotz , E. R. Jones , H. R. Screen , and G. P. Riley , “Increased Expression of IL‐6 Family Members in Tendon Pathology,” Rheumatology 51, no. 7 (2012): 1161–1165. 22337942 10.1093/rheumatology/kes002 PMC3380247

[75] F. Klatte‐Schulz , S. Minkwitz , A. Schmock , et al., “Different Achilles Tendon Pathologies Show Distinct Histological and Molecular Characteristics,” International Journal of Molecular Sciences 19, no. 2 (2018): 404. 29385715 10.3390/ijms19020404 PMC5855626

[76] R. Abiola , P. Rubery , and A. Mesfin , “Ossification of the Posterior Longitudinal Ligament: Etiology, Diagnosis, and Outcomes of Nonoperative and Operative Management,” Global Spine Journal 6, no. 2 (2016): 195–204. 26933622 10.1055/s-0035-1556580 PMC4771496

[77] B. W. Choi , K. J. Song , and H. Chang , “Ossification of the Posterior Longitudinal Ligament: A Review of Literature,” Asian Spine Journal 5, no. 4 (2011): 267–276. 22164324 10.4184/asj.2011.5.4.267 PMC3230657

[78] T. Hirai , T. Yoshii , A. Iwanami , et al., “Prevalence and Distribution of Ossified Lesions in the Whole Spine of Patients With Cervical Ossification of the Posterior Longitudinal Ligament A Multicenter Study (JOSL CT Study),” PLoS One 11, no. 8 (2016): e0160117. 27548354 10.1371/journal.pone.0160117 PMC4993375

[79] Y. Shirakura , T. Sugiyama , H. Tanaka , T. Taguchi , and S. Kawai , “Hyperleptinemia in Female Patients With Ossification of Spinal Ligaments,” Biochemical and Biophysical Research Communications 267 (2000): 752–755. 10673363 10.1006/bbrc.1999.2027

[80] B. Feng , S. Cao , J. Zhai , et al., “Roles and Mechanisms of Leptin in Osteogenic Stimulation in Cervical Ossification of the Posterior Longitudinal Ligament,” Journal of Orthopaedic Surgery and Research 13 (2018): 165. 29970120 10.1186/s13018-018-0864-4 PMC6029428

[81] Y. Ikeda , A. Nakajima , A. Aiba , et al., “Association Between Serum Leptin and Bone Metabolic Markers, and the Development of Heterotopic Ossification of the Spinal Ligament in Female Patients With Ossification of the Posterior Longitudinal Ligament,” European Spine Journal 20 (2011): 1450–1458. 21258825 10.1007/s00586-011-1688-7 PMC3175891

[82] S. Tenti , P. Palmitesta , N. Giordano , M. Galeazzi , and A. Fioravanti , “Increased Serum Leptin and Visfatin Levels in Patients With Diffuse Idiopathic Skeletal Hyperostosis: A Comparative Study,” Scandinavian Journal of Rheumatology 46, no. 2 (2017): 156–158. 27684733 10.1080/03009742.2016.1188981

[83] R. Mader , I. Novofastovski , N. Schwartz , and E. Rosner , “Serum Adiponectin Levels in Patients With Diffuse Idiopathic Skeletal Hyperostosis (DISH),” Clinical Rheumatology 37, no. 10 (2018): 2839–2845. 30121711 10.1007/s10067-018-4258-0

[84] F. Fang , L. Liu , Y. Yang , et al., “The Adipokine Adiponectin Has Potent Anti‐Fibrotic Effects Mediated via Adenosine Monophosphate‐Activated Protein Kinase: Novel Target for Fibrosis Therapy,” Arthritis Research & Therapy 14 (2012): R229. 23092446 10.1186/ar4070 PMC3580540

[85] K. Lakota , J. Wei , M. Carns , et al., “Levels of Adiponectin, a Marker for PPAR‐Gamma Activity, Correlate With Skin Fibrosis in Systemic Sclerosis: Potential Utility as a Biomarker?,” Arthritis Research & Therapy 14 (2012): R102–R110. 22548780 10.1186/ar3827 PMC3446479

[86] R. H. Storkson , R. Aamodt , K. K. Vetvik , et al., “mRNA Expression of Adipocytokines and Glucocorticoid‐Related Genes Are Associated With Downregulation of E‐Cadherin mRNA in Colorectal Adenocarcinomas,” International Journal of Colorectal Disease 27 (2012): 1021–1027. 22411584 10.1007/s00384-012-1442-6

[87] M. R. Schmidli , B. Fuhrer , N. Kurt , et al., “Inflammatory Pattern of the Infrapatellar Fat Pad in Dogs With Canine Cruciate Ligament Disease,” BMC Veterinary Research 14 (2018): 161. 29769086 10.1186/s12917-018-1488-y PMC5956839

[88] G. J. Farkas , A. S. Gorgey , D. R. Dolbow , A. S. Berg , and D. R. Gater , “The Influence of Level of Spinal Cord Injury on Adipose Tissue and Its Relationship to Inflammatory Adipokines and Cardiometabolic Profiles,” Journal of Spinal Cord Medicine 41, no. 4 (2018): 407–415. 28758566 10.1080/10790268.2017.1357918 PMC6055972

[89] G. G. Whiteneck , S. W. Charlifue , H. L. Frankel , et al., “Mortality, Morbidity, and Psychosocial Outcomes of Persons Spinal Cord Injured More Than 20 Years Ago,” Paraplegia 30, no. 9 (1992): 617–630. 1408338 10.1038/sc.1992.124

[90] J. D. Chamberlain , S. Meier , L. Mader , P. M. von Groote , and M. W. Brinkhof , “Mortality and Longevity After a Spinal Cord Injury: Systematic Review and Meta‐Analysis,” Neuroepidemiology 44, no. 3 (2015): 182–198. 25997873 10.1159/000382079

[91] D. R. Gater, Jr. , G. J. Farkas , and E. Tiozzo , “Pathophysiology of Neurogenic Obesity After Spinal Cord Injury,” Top Spinal Cord Inj Rehabil 27, no. 1 (2021): 1–10. 10.46292/sci20-00067 PMC7983633 33814879

[92] G. J. Farkas and D. R. Gater , “Neurogenic Obesity and Systemic Inflammation Following Spinal Cord Injury: A Review,” Journal of Spinal Cord Medicine 41, no. 4 (2018): 378–387. 28758554 10.1080/10790268.2017.1357104 PMC6055969

[93] T. H. Lee , K. K. Cheng , R. L. Hoo , P. M. Siu , and S. Y. Yau , “The Novel Perspectives of Adipokines on Brain Health,” International Journal of Molecular Sciences 20, no. 22 (2019): 5638. 31718027 10.3390/ijms20225638 PMC6887733

[94] R. Brown , S. A. Imran , D. D. Belsham , E. Ur , and M. Wilkinson , “Adipokine Gene Expression in a Novel Hypothalamic Neuronal Cell Line: Resistin‐Dependent Regulation of Fasting‐Induced Adipose Factor and SOCS‐3,” Neuroendocrinology 85, no. 4 (2007): 232–241. 17579277 10.1159/000104248

[95] M. Wilkinson , R. Brown , S. A. Imran , and E. Ur , “Adipokine Gene Expression in Brain and Pituitary Gland,” Neuroendocrinology 86, no. 3 (2007): 191–209. 17878708 10.1159/000108635

[96] S. Latifi , D. Koushki , A. Norouzi Javidan , M. Matin , and H. Sabour , “Changes of Leptin Concentration in Plasma in Patients With Spinal Cord Injury: A Meta‐Analysis,” Spinal Cord 51 (2013): 728–731. 23999108 10.1038/sc.2013.82

[97] G. E. Bigford , V. C. Bracchi‐Ricard , M. S. Nash , and J. R. Bethea , “Alterations in Mouse Hypothalamic Adipokine Gene Expression and Leptin Signaling Following Chronic Spinal Cord Injury and With Advanced Age,” PLoS One 7, no. 7 (2012): e41073. 22815920 10.1371/journal.pone.0041073 PMC3397960

[98] A. L. Doherty , R. A. Battaglino , J. Donovan , et al., “Adiponectin Is a Candidate Biomarker of Lower Extremity Bone Density in Men With Chronic Spinal Cord Injury,” Journal of Bone and Mineral Research 29, no. 1 (2014): 251–259. 23787489 10.1002/jbmr.2020 PMC3979427

[99] C. O. Tan , R. A. Battaglino , A. L. Doherty , et al., “Adiponectin Is Associated With Bone Strength and Fracture History in Paralyzed Men With Spinal Cord Injury,” Osteoporosis International 25, no. 11 (2014): 2599–2607. 24980185 10.1007/s00198-014-2786-2 PMC4861654

[100] M. P. Sullivan , S. J. Torres , S. Mehta , and J. Ahn , “Heterotopic Ossification After Central Nervous System Trauma: A Current Review,” Bone & Joint Research 2, no. 3 (2013): 51–57. 23610702 10.1302/2046-3758.23.2000152 PMC3626201

[101] G. Lutchman , K. Promrat , D. E. Kleiner , et al., “Changes in Serum Adipokine Levels During Pioglitazone Treatment for Nonalcoholic Steatohepatitis: Relationship to Histological Improvement,” Clinical Gastroenterology and Hepatology 4, no. 8 (2006): 1048–1052. 16814613 10.1016/j.cgh.2006.05.005

[102] Y. Saitoh , C. Chun‐ping , K. Noma , H. Ueno , M. Mizuta , and M. Nakazato , “Pioglitazone Attenuates Fatty Acid‐Induced Oxidative Stress and Apoptosis in Pancreatic Beta‐Cells,” Diabetes, Obesity & Metabolism 10, no. 7 (2008): 564–573. 10.1111/j.1463-1326.2007.00749.x 17593232

[103] Y. Hu , L. Huang , M. Shen , et al., “Pioglitazone Protects Compression‐Mediated Apoptosis in Nucleus Pulposus Mesenchymal Stem Cells by Suppressing Oxidative Stress,” Oxidative Medicine and Cellular Longevity 2019 (2019): 4764071. 31885796 10.1155/2019/4764071 PMC6893265

[104] D. Chen , D. Xia , Z. Pan , et al., “Metformin Protects Against Apoptosis and Senescence in Nucleus Pulposus Cells and Ameliorates Disc Degeneration In Vivo,” Cell Death & Disease 7 (2016): e2441. 27787519 10.1038/cddis.2016.334 PMC5133996

[105] M. Yao , J. Zhang , Z. Li , X. Bai , J. Ma , and Y. Li , “Liraglutide Protects Nucleus Pulposus Cells Against High‐Glucose Induced Apoptosis by Activating PI3K/Akt/ mTOR/Caspase‐3 and PI3K/Akt/GSK3β/Caspase‐3 Signaling Pathways,” Front Med (Lausanne) 8 (2021): 630962. 33681258 10.3389/fmed.2021.630962 PMC7933515

[106] N. Adamia , D. Virsaladze , N. Charkviani , M. Skhirtladze , and M. Khutsishvili , “Effect of Metformin Therapy on Plasma Adiponectin and Leptin Levels in Obese and Insulin Resistant Postmenopausal Females With Type 2 Diabetes,” Georgian Medical News 145 (2007): 52–55. 17525501

[107] P. V. Dludla , B. B. Nkambule , S. E. Mazibuko‐Mbeje , et al., “Adipokines as a Therapeutic Target by Metformin to Improve Metabolic Function: A Systematic Review of Randomized Controlled Trials,” Pharmacological Research 163 (2021): 105219. 33017649 10.1016/j.phrs.2020.105219

[108] D. Graf , S. Seifert , A. Jaudszus , A. Bub , and B. Watzl , “Anthocyanin‐Rich Juice Lowers Serum Cholesterol, Leptin, and Resistin and Improves Plasma Fatty Acid Composition in Fischer Rats,” PLoS One 8, no. 6 (2013): e66690. 23825152 10.1371/journal.pone.0066690 PMC3688949

[109] A. Luvizotto Rde , A. F. Nascimento , E. Imaizumi , et al., “Lycopene Supplementation Modulates Plasma Concentrations and Epididymal Adipose Tissue mRNA of Leptin, Resistin and IL‐6 in Diet‐Induced Obese Rats,” British Journal of Nutrition 110, no. 10 (2013): 1803–1809. 23632237 10.1017/S0007114513001256

[110] S. O. Cho , M. H. Kim , and H. Kim , “β‐Carotene Inhibits Activation of NF‐κB, Activator Protein‐1, and STAT3 and Regulates Abnormal Expression of Some Adipokines in 3T3‐L1 Adipocytes,” Journal of Cancer Prevention 23, no. 1 (2018): 37–43. 29629347 10.15430/JCP.2018.23.1.37 PMC5886493

[111] S. Khateeb , A. Albalawi , and A. Alkhedaide , “Diosgenin Modulates Oxidative Stress and Inflammation in High‐Fat Diet‐Induced Obesity in Mice,” Diabetes, Metabolic Syndrome and Obesity 15 (2022): 1589–1596. 10.2147/DMSO.S355677 PMC9147404 35637860

[112] Y. Chen , X. Xu , Y. Zhang , et al., “Diosgenin Regulates Adipokine Expression in Perivascular Adipose Tissue and Ameliorates Endothelial Dysfunction via Regulation of AMPK,” Journal of Steroid Biochemistry and Molecular Biology 155, no. Pt A (2016): 155–165. 26277096 10.1016/j.jsbmb.2015.07.005

[113] J. Olholm , S. K. Paulsen , K. B. Cullberg , B. Richelsen , and S. B. Pedersen , “Anti‐Inflammatory Effect of Resveratrol on Adipokine Expression and Secretion in Human Adipose Tissue Explants,” International Journal of Obesity 34, no. 10 (2010): 1546–1553. 20531350 10.1038/ijo.2010.98

[114] J. F. Landrier , E. Gouranton , C. El Yazidi , et al., “Adiponectin Expression Is Induced by Vitamin E via a Peroxisome Proliferator‐Activated Receptor Gamma‐Dependent Mechanism,” Endocrinology 150, no. 12 (2009): 5318–5325. 19833717 10.1210/en.2009-0506

[115] F. S. Lira , J. C. Rosa , C. A. Cunha , et al., “Supplementing Alpha‐Tocopherol (Vitamin E) and Vitamin D3 in High Fat Diet Decrease IL‐6 Production in Murine Epididymal Adipose Tissue and 3T3‐L1 Adipocytes Following LPS Stimulation,” Lipids in Health and Disease 10 (2011): 37. 21352586 10.1186/1476-511X-10-37 PMC3050762

[116] S. Pummoung , D. Werawatganon , M. Chayanupatkul , N. Klaikeaw , and P. Siriviriyakul , “Genistein Modulated Lipid Metabolism, Hepatic PPARγ, and Adiponectin Expression in Bilateral Ovariectomized Rats With Nonalcoholic Steatohepatitis (NASH),” Antioxidants (Basel) 10, no. 1 (2020): 24. 33383845 10.3390/antiox10010024 PMC7824652

[117] Y. Panahi , M. S. Hosseini , N. Khalili , et al., “Effects of Supplementation With Curcumin on Serum Adipokine Concentrations: A Randomized Controlled Trial,” Nutrition 32, no. 10 (2016): 1116–1122. 27297718 10.1016/j.nut.2016.03.018

[118] A. K. Lederer , M. A. Storz , R. Huber , L. Hannibal , and E. Neumann , “Plasma Leptin and Adiponectin After a 4‐Week Vegan Diet: A Randomized‐Controlled Pilot Trial in Healthy Participants,” International Journal of Environmental Research and Public Health 19, no. 18 (2022): 11370. 36141644 10.3390/ijerph191811370 PMC9517500

[119] J. Ambroszkiewicz , J. Gajewska , J. Mazur , et al., “Does a Vegetarian Diet Affect the Levels of Myokine and Adipokine in Prepubertal Children?,” Journal of Clinical Medicine 10, no. 17 (2021): 3995. 34501443 10.3390/jcm10173995 PMC8432473

[120] M. Vučić Lovrenčić , M. Gerić , I. Košuta , M. Dragičević , V. Garaj‐Vrhovac , and G. Gajski , “Sex‐Specific Effects of Vegetarian Diet on Adiponectin Levels and Insulin Sensitivity in Healthy Non‐Obese Individuals,” Nutrition 79‐80 (2020): 110862. 10.1016/j.nut.2020.110862 32711387

